# Quality of relationships in mothers and their partners in the Avon Longitudinal Study of Parents and Children

**DOI:** 10.12688/wellcomeopenres.18835.1

**Published:** 2023-02-06

**Authors:** Hamid Reza Tohidinik, Yoav Ben-Shlomo, Daniel Major-Smith, Neil Goulding, Yasmin Iles-Caven, Jean Golding, Kate Northstone, Abigail Fraser

**Affiliations:** 1 Centre for Academic Child Health, Population Health Sciences, Bristol Medical School, University of Bristol, Bristol, BS8 2BN, UK; 2 Population Health Sciences, Bristol Medical School, Bristol, BS8 2PS, UK

**Keywords:** Relationship quality, partners, ALSPAC, Intimate Bond Measure, Care, Control, Spouse, longitudinal

## Abstract

Quality of relationship between partners is associated with a wide range of physical and psychological outcomes like anxiety and depression. There are relatively few longitudinal studies with detailed and repeated measures for quality of relationship, particularly in both partners. The Avon Longitudinal Study of Parents and Children (ALSPAC) is a large birth cohort study in the UK with five post-partum repeated measures of quality of relationship between mothers and their partners assessed using the Intimate Bond Measure (IBM). The Measure includes two subscales named “Care” and “Control”. These were measured at 2.75, 6, 9, 12, and 18 years post-partum (baseline N for mothers: 8675; baseline N for partners: 5499). The aims of this data note are to provide a comprehensive overview on the existing IBM data in ALSPAC and to describe both its strengths and limitations for future users. The internal consistency of the subscales were high (Cronbach's alpha 0.95 and 0.88 for the Care and Control subscales) in both mothers and their partners at the baseline. In the Care subscale, all 12 items were highly correlated with the overall score (r>0.62) at the baseline, but in the Control subscale there were three items that had relatively low correlations with the total subscale (r<0.46). This should be taken into account in future research. The longitudinal nature of this data on both mothers and partners will enable detailed explorations of the causes and consequences of differences in quality of relationship.

## Introduction

Many studies in social epidemiology have examined whether psychosocial factors are important in determining health outcomes either directly or as mediators that may partially explain social inequalities. One such domain has been the broad area of social support ( Taylor, 2011). In the classic Alameda county study in California, men and women who were socially isolated were over twice as likely to die than those with good social networks and the main determinant of good support was having an intimate partner ( Berkman & Syme, 1979). This has led to interest in how intimate partner relationships and more importantly the quality of such a relationship can influence health either through psychoneuroendocrine mechanisms or indirectly through better health behaviours or increased socioeconomic status ( Uchino, 2006).

Quality of relationship (QoR) in intimate partners is associated with a wide range of physical and psychological outcomes, including depression and anxiety ( Frech & Williams, 2007; Holt-Lunstad *et al.*, 2008; Pieh *et al.*, 2020), sleep quality ( Pieh *et al.*, 2020), and a range of cardiovascular diseases (CVD) risk factors ( Bennett-Britton *et al.*, 2017; Holt-Lunstad *et al.*, 2008). Most published studies regarding QoR and health outcomes are cross-sectional ( Eder *et al.*, 2021; Pieh *et al.*, 2020) and are subject to various biases and issues with reverse causation ( Rothman, 2002). For instance, rather than QoR causing these health outcomes, perhaps these health conditions put a strain on relationships, worsening QoR. QoR does not remain constant over time ( Bennett-Britton *et al.*, 2017) and cross-sectional approaches cannot capture this. Therefore, longitudinal studies that assess QoR over time are important, especially when attempting to establish causality. Such studies are also useful tools to identify determinants of QoR changes over time and to examine its role as a predictor for other health outcomes ( Hernán & Robins, 2020).

The Avon Longitudinal Study of Parents and Children (ALSPAC) is a longitudinal birth cohort with a wide range of genetic, psychological, physical, and environmental data ( Boyd *et al.*, 2013; Fraser *et al.*, 2013; Golding *et al.*, 2001). QoR has been assessed in both mothers and their partners using the Intimate Bond Measure (IBM) ( Wilhelm & Parker, 1988), at five different time points over a twenty-year period. The aims of this data note are to provide a comprehensive overview of the existing IBM data and to describe both its strengths and limitations thereby helping future researchers who may wish to use it to test their own hypotheses.

## Methods

### The ALSPAC sample

ALSPAC is a longitudinal birth cohort in which all pregnant women resident in the Bristol area, UK with expected dates of delivery between 1st April 1991 and 31st December 1992, were eligible to join the study. The initial number of enrolled pregnancies was 14,541, resulting in 14,062 live births and 13,988 children who were alive by the age of 1 year.

Of the original 14,541 initial pregnancies, 338 were from a woman who had already enrolled with a previous pregnancy, meaning 14,203 unique mothers were initially enrolled in the study. As a result of the additional phases of recruitment, a further 630 women who did not enrol originally have provided data since their child was 7 years of age. This provides a total of 14,833 unique women (G0 mothers) enrolled in ALSPAC as of September 2021 ( Major-Smith *et al.*, 2022).

G0 partners were invited to complete questionnaires by the mothers at the start of the study and they were not formally enrolled at that time. 12,113 G0 partners have been in contact with the study by providing data and/or formally enrolling when this started in 2010. 3,807 G0 partners are currently enrolled ( Northstone *et al.*, 2023).

The mothers, their partners, and the index children have been followed-up and data on sociodemographic, physical, biological, mental, and behavioural factors have been gathered using self-completion questionnaires, clinical assessments and links to routine data. It is important to note that we have only included mothers who had partners and were living with their partners when completing the questionnaires. Partners can and will change over time so that different individuals may complete the partner questionnaire over time. The ALSPAC team has recently derived and made available variables that capture changes in ALSPAC mothers’ partners over time.

Further details on the ALSPAC study have been published elsewhere ( Boyd *et al.*, 2013; Fraser *et al.*, 2013; Golding *et al.*, 2001; Northstone *et al.*, 2019) The study website contains details of all data available through a fully searchable data dictionary and variable search tool: http://www.bristol.ac.uk/alspac/researchers/our-data/.

### Quality of relationship

***The Intimate Bond Measure (IBM).*** QoR was assessed using the 24-item Intimate Bond Measure (IBM) ( Wilhelm & Parker, 1988), which includes two separate 12-item subscales named (a) Care - indicating the level of kindness and warmth shown by the respondent’s partner and (b) Control - indicating the level of criticism and dominance shown by the respondent’s partner.

The IBM was included in questionnaires sent to ALSPAC mothers and their partners (G0s) at five timepoints. Four timepoints were the same in both parents: at 2 years 9 months after the birth of the index child, at 6 years 1 month, 9 years 2 months, and at 12 years 1 month. The fifth questionnaire was completed at 18 years 5 months by G0 mothers and at 20 years 5 months by their partners. The list of IBM items along with their corresponding variable names in the ALSPAC data files is available in Supplemental Table 1 to assist potential users.

***Scoring and deriving the IBM subscales***. The 24 IBM related questions were recoded from 1, 2, 3, 4 to 3, 2, 1, 0 for Very true, Mostly true, Somewhat true, and Not at all true, respectively, to make them compatible with the original questionnaire scoring ( Table 1) ( Wilhelm & Parker, 1988).

**Table 1.** Characteristics of G0 respondents to questionnaires including the IBM at different timepoints in ALSPAC †.

Variables	Time points (age of index children)									
	2 years 9 months		6 years 1 months		9 years 2 months		12 years 1 months		18 years 5 months	20 years 5 months
	Mothers	Partners	Mothers	Partners	Mothers	Partners	Mothers	Partners	Mothers	Partners
**No. sent questionnaires**	13968	12085	10869	10282	11137	8001	10171	7091	9034	-
**No. returned questionnaires** (response rate) *	9618 (68.8%)	5449 (45.1%)	8513 (78.3%)	4459 (43.4%)	7965 (71.5%)	3662 (45.8%)	7034 (69.2%)	3295 (46.5%)	4170 (46.2%)	2469
**Who completed the** ** questionnaire, N (%) ¥**										
Mother only	9421 (97.95)	65 (1.19)	8374 (98.37)	<5	7791 (97.82)	<5	6859 (97.52)	<5	4069 (97.58)	<5
Father only **	18 (0.19)	5096 (93.52)	9 (0.11)	4179 (93.72)	<5	3581 (97.78)	<5	3223 (97.81)	<5	2382
Other combinations †	46 (0.48)	104 (1.90)	28 (0.32)	170 (3.81)	93 (1.16)	27 (0.74)	122 (1.7)	38 (1.15)	46 (1.11)	13
Missing	133 (1.38)	184 (3.38)	102 (1.20)	110 (2.47)	81 (1.02)	54 (1.48)	53 (0.76)	34 (1.03)	55 (1.31)	74
**Has a partner, N (%)**										
Male partner	8,906 (92.60)	-	7865 (92.39)	4366 (97.91)	7280 (91.4)	<5	6340 (90.14)	<5	-	-
Female partner	39 (0.41)	-	20 (0.23)		9 (0.11)	3548 (96.90)	14 (0.2)	3194 (96.93)	-	-
No partner	608 (6.32)	-	574 (6.74)	44 (0.99)	620 (7.78)	69 (1.87)	641 (9.11)	84 (2.55)	-	-
Not stated	65 (0.68)	-	54 (0.63)	49 (1.1)	56 (0.71)	45 (1.23)	39 (0.55)	16 (0.49)	-	-
**Living with a partner, N (%)**										
Yes	8,675 (90.20)	-	7516 (88.29)	3975 (89.15)	6921 (86.89)	3491 (95.33)	5993 (85.20)	3136 (95.17)	3345 (80.22)	2095 (84.85)
No	264 (2.74)	-	382(4.49)	326 (7.31)	379 (4.76)	51 (1.39)	356 (5.06)	54 (1.64)	710 (17.03)	175 (7.09)
Not stated	71 (0.74)	-	615 (7.22)	158 (3.54)	665 (8.35)	120 (3.28)	685 (9.74)	105 (3.19)	115 (2.76)	199 (8.06)

*If the questionnaire was returned blank, we considered it as not returned; **both biological and step-fathers † Mother & father, Mother & other, Father & other, other only †At time of writing (March 2022), 22 mothers and 5 partners had withdrawn consent for their data to be used, and so are excluded from the numbers reported here. ¥ Only among those who returned the questionnaire. Eight “<5” cells include zero.

As each question is scored between 0–3, and the number of questions for each subscale is 12, the resulting summary score of all items could range between 0–36, with higher scores indicating greater Care (positive attribute of relationship) and greater Control (usually regarded as a negative attribute of relationship) ( Wilhelm & Parker, 1988).

***Internal consistency and deriving subscale scores.*** Internal consistency of the IBM subscales was assessed using Cronbach’s Alpha. We also assessed the correlation between each item and the total subscale score without that item (item-rest correlation) to see how well each item measures the same construct as the other items in combination. This quantifies the relative importance of any missing data for a given item when deriving the total subscale score. On the basis of these results we formulated our approach to derive a score for participants where one or more specific items are missing to avoid loss of data and the need to undertake multiple imputation, for the purpose of this Data Note.

***Distributions of Care and Control scores and associations with sociodemographic variables.*** We examined the distributions of the subscale scores to check whether assumptions of normality were reasonable or not and whether specific transformations may be of value to enable parametric analyses. We also examined the associations between the Care and Control subscales with age, education level, occupational social class and parity to look for patterns that may provide some form of external validity. The Cuzick test of trend was used to identify any trend across groups. The associations between sociodemographic variables and missingness in IBM were assessed using logistic regression. All statistical analyses were performed separately in mothers and their partners using Stata 17.0 (StataCorp LLC, College Station, TX, USA).

## Results

The number of G0 mothers and partners who returned the questionnaires varied from 9,638 and 5,499 respectively, at baseline (age of index child: 2 years 9 months; mean±Standard Deviation [SD], maternal age: 32.27±4.59; mean±SD partners’ age: 34.29±5.63) to 4,170 and 2,469 at timepoint 5 (age of index child: 18 years 5 months to 20 years 5 months; mean±SD maternal age: 48.62±4.40; mean±SD partners’ age: 53.30±5.36). The number of mothers living with their partners declined from 8,675 (90.0%) at baseline to 3,345 (80.2%) at timepoint 5 ( Table 1) amongst mothers who returned the questionnaire.

### Pattern of missing IBM items

Determinants of loss to follow up in ALSPAC have been previously identified ( Howe *et al.*, 2013). Missing data on the IBM may arise for different reasons (i) drop out of Study; (ii) no correct contact address (iii) non-return of the questionnaire or (iv) return of questionnaire but missing data on part or all of the IBM. Here we present the extent of missing IBM data in participants who returned the relevant questionnaires and were living with their partners. As can be seen from Table 2a– Table 2e, the extent of missing data was low for the IBM subscales at the five timepoints. The minimum proportion of mothers who completed all IBM items were 95.1% and 93.1% for the Care and Control subscales, respectively. All-item missingness (i.e. the questionnaire was returned but none of the IBM items were completed) did not exceed 1.9% and 1.9% for the Care and Control subscales, respectively. At all timepoints the proportion of partners who returned the questionnaire and completed all IBM items were more than 96.3% and 94.7%, respectively, whereas the maximum all-item missingness were 1.6% and 1.7% for the Care and Control subscales, respectively. There was no relation between at least one item missingness and sociodemographic variables such as age, occupational social class, and Index of Multiple Deprivation (IMD). However, higher education was associated with lower chance of at least one item missingness in both mothers and their partners (Supplemental Table 2).

**Table 2a.** Pattern of missing data for IBM subscales at baseline (2 years 9 months) in G0 mothers and their partners in ALSPAC.

	Mothers, N=8675 *		Partners, N=5499	
Number of missing items	Care, N (%)	Control, N (%)	Care, N (%)	Control, N (%)
0	8372 (96.5%)	8213 (94.7%)	5295 (96.3%)	5207 (94.7%)
1	142	257	68	156
2	17	40	5	6
3	9	19	7	<5
4	25	32	<5	6
5	<5	7	<5	<5
6	<5	<5	<5	<5
7	5	<5	<5	<5
8	29	30	<5	<5
9	<5	<5	<5	<5
10	5	<5	<5	<5
11	5	<5	<5	<5
12	61 (0.7%)	65 (0.75%)	61 (1.12%)	63 (1.11%)
Which question was the most common missing [No. Missing (%)]	H627: Partner is gentle and kind to me [134 (1.54)]	H606: Partner wants me to take his/her side in an argument [224 (2.58)]	pf6130: Partner shows his/her appreciation of me [91 (1.65)]	pf6131: Partner is critical of me in private [126 (2.29)]

*Only mothers who lived with their partners. Three “<5” cells include zero.

**Table 2b.** Pattern of missing data for IBM subscales at timepoint 2 (6 years 1 month) in G0 mothers and their partners in ALSPAC.

	Mothers, N=7516 *		Partners, N= 3975 *	
Number of missing items	Care, N (%)	Control, N (%)	Care, N (%)	Control, N (%)
0	7284 (96.9%)	7134 (94.9%)	3901 (98.1%)	3834 (96.4%)
1	93	222	34	94
2	18	50	11	17
3	28	13	<5	<5
4	<5	6	<5	<5
5	<5	<5	<5	<5
6	<5	<5	<5	<5
7	<5	<5	<5	<5
8	<5	7	<5	<5
9	18	<5	<5	<5
10	<5	13	<5	<5
11	7	<5	<5	<5
12	53 (0.7%)	60 (0.8%)	23 (0.58%)	22 (0.55%)
Which question was the most common missing [No. Missing (%)]	L6207: Degree to which respondent's partner confides closely in [112 (1.49)]	L6201: Partner wants me to take his/her side in an argument [193 (2.57)]	pj6207: Degree to which respondent's partner confides closely in [39 (0.98)]	pj6221: Partner is critical of me in private [73 (1.84)]

*Only participants who lived with their partners. Eleven “<5” cells include zero.

**Table 2c.** Pattern of missing data for IBM subscales at timepoint 3 (9 years 2 months) in G0 mothers and their partners in ALSPAC.

	Mothers, N=6921 *		Partners, N=3491 *	
Number of missing items	Care, N (%)	Control, N (%)	Care, N (%)	Control, N (%)
0	6584 (95.1%)	6442 (93.1%)	3386 (97.0%)	3,335 (95.5%)
1	91	206	31	73
2	12	34	5	16
3	8	9	<5	<5
4	<5	5	<5	<5
5	7	44	<5	<5
6	112	<5	6	<5
7	6	72	0	5
8	<5	6	<5	<5
9	<5	<5	<5	<5
10	<5	<5	<5	<5
11	<5	<5	<5	<5
12	86 (1.24%)	91 (1.31%)	54 (1.55%)	55 (1.58%)
Which question was the most common missing [No. Missing (%)]	p3212: Partner is physically gentle and considerate [201 (2.90)]	P3201: Partner wants me to take his/her side in an argument [286 (4.13)]	pm3212: Partner is physically gentle and considerate [74 (2.12)]	pm3201: Partner wants me to take his/her side in an argument [100 (2.86)]

*Only participants who lived with their partners. Seven “<5” cells include zero.

**Table 2d.** Pattern of missing data for IBM subscales at timepoint 4 (12 years 1 month) in G0 mothers and their partners in ALSPAC.

	Mothers, N=5993 *		Partners, N=3136 *	
Number of missing items	Care, N (%)	Control, N (%)	Care, N (%)	Control, N (%)
0	5741 (97.8)	5635 (94.0%)	3049 (97.2%)	2991 (95.4%)
1	82	173	28	82
2	22	25	6	5
3	8	13	<5	<5
4	<5	6	<5	<5
5	<5	6	<5	7
6	12	5	11	<5
7	<5	9	<5	7
8	<5	<5	<5	<5
9	<5	<5	<5	<5
10	<5	<5	<5	<5
11	5	5	<5	<5
12	111 (1.85%)	112 (1.87%)	39 (1.24%)	41 (1.31%)
Which question was the most common missing [No. Missing (%)]	s3212: Partner is physically gentle and considerate [162 (2.75)]	S3201: Partner wants me to take his/her side in an argument [228 (3.80)]	pq3207: Partner confides closely in me [56 (1.79)] pq3209: Understands my problems and worries [56 (1.79)]	pq3221: Partner is critical of me in private [91 (2.90)]

*Only participants who lived with their partners. Twelve “<5” cells include zero.

**Table 2e.** Pattern of missing data for IBM subscales at timepoint 5 (18 y 5 m) in G0 mothers and their partners in ALSPAC.

	Mothers, N=3345 *		Partners, N=2095 *	
Number of missing items	Care, N (%)	Control, N (%)	Care, N (%)	Control, N (%)
0	3239 (96.8%)	3196 (95.5%)	2032 (97.0%)	1987 (94.8%)
1	58	90	27	73
2	5	10	<5	<5
3	<5	7	<5	<5
4	5	<5	<5	<5
5	<5	<5	<5	<5
6	<5	<5	<5	<5
7	<5	<5	<5	<5
8	<5	<5	<5	<5
9	<5	<5	<5	<5
10	<5	<5	<5	<5
11	<5	<5	<5	<5
12	33 (0.99%)	34 (1.02%)	20 (0.95%)	22 (1.05%)
Which question was the most common missing [No. Missing (%)]	t1103: Partner is good companion [55 (1.64)]	t1101: Partner wants me to take his/her side in an argument [71 (2.12)]	Fa1117: Partner is fun to be with [34 (1.62)]	fa1121: Partner is critical of me in private [62 (2.96)]

*Only participants who lived with their partners. Fifteen “<5” cells include zero.

The items most frequently not answered by participants returning the questionnaires were:

Mothers:

Care: Partner is physically gentle and considerate (h617, l6212, p3212, s3212, t1112).

Control: Partner wants me to take his/her side in an argument (h606, l6201, p3201, s3201, t1101).

Partners:

Care: Partner confides closely in me (pf6117, pj6207, pm3207, pq3207, fa1107).

Control: Partner is critical of me in private (pf6131, pj6221, pm3221, pq3221, fa1121).

### Subscale score generation

The correlations between each item for the Care and Control subscales and the overall score at the baseline (2.75 years postpartum) are presented in Table 3 & Table 4 for mothers and partners respectively. For the Care subscale, all items were highly correlated to the overall score with the minimum correlation for item 8 (‘my partner confides closely in me’) that was 0.63 for mothers and 0.62 for their partners. This indicates that all items in the Care subscale are strongly associated with this dimension, meaning that all items can be treated equally, regardless of missingness status. Here we included participants with up to 6 missing items in the Care subscale and weighted the completed responses as if all 12 items had been completed. Thus, the total score of cases with 1, 2, 3, 4, 5, and 6 missing items can be multiplied by 1.09, 1.20, 1.33, 1.50, 1.71, and 2.00, respectively.

**Table 3.** Internal consistency and item-related characteristics for IBM “Care” and “Control” subscales among G0 mothers in ALSPAC *.

Item in ALSPAC	Questions	No. Observations ^†^	Item-rest correlation	Cronbach’s alpha if item deleted
**Care subscale**	My partner...			
**h605**	1. Is very considerate of me	8570	0.75	0.95
**h608**	4. Is a good companion	8565	0.77	0.95
**h609**	5. Is affectionate to me	8568	0.79	0.95
**h612**	8. Confides closely in me	8557	0.63	0.95
**h614**	10. Understands my problems and worries	8553	0.68	0.95
**h617**	13. Is physically gentle and considerate	8546	0.73	0.95
**h618**	14. Makes me feel needed	8550	0.81	0.94
**h620**	16. Is very loving to me	8559	0.83	0.94
**h622**	18. Is fun to be with	8550	0.76	0.95
**h625**	21. Show his/her appreciation of me	8545	0.77	0.95
**h627**	23. Is gentle and kind to me	8541	0.84	0.94
**h628**	24. Speaks to me in a warm and friendly voice	8554	0.77	0.95
**Control subscale**				
**h606**	2. Wants me to take his/her side in an argument	8451	0.36	0.89
**h607**	3. Wants to know exactly what I'm doing and where I am	8556	0.40	0.88
**h610**	6. Is clearly hurt if I don't accept his/her views	8532	0.46	0.88
**h611**	7. Tends to try and change me	8555	0.71	0.87
**h613**	9. Tends to criticize me over small issues	8558	0.63	0.87
**h615**	11. Tends to order me about	8566	0.71	0.87
**h616**	12. Insists I do exactly as I'm told	8562	0.64	0.87
**h619**	15. Wants me to change in small ways	8533	0.54	0.88
**h621**	17. Seeks to dominate me	8529	0.71	0.87
**h623**	19. Wants to change me in big ways	8544	0.62	0.87
**h624**	20 Tends to control everything I do	8545	0.62	0.87
**h626**	22. Is critical of me in private	8493	0.59	0.87
**Total Cronbach’s alpha for Care and Control subscales were 0.95 and 0.88, respectively.**				

IBM: Intimate Bond Measure; ALSPAC: Avon Longitudinal Study of Parents and Children *It is measured only for mothers who lived with their partners when the age of index children was 2 years 9 months (h questionnaire). **†**The number of non missing values of the item.

**Table 4.** Internal consistency and item-related characteristics for IBM “Care” and “Control” subscales among partners of G0 mothers in ALSPAC *.

Item in ALSPAC	Questions	No. observations †	Item-rest correlation	Cronbach’s alpha if item deleted
**Care subscale**	My partner...			
**pf6110**	1. Is very considerate of me	5366	0.74	0.95
**pf6113**	4. Is a good companion	5373	0.73	0.95
**pf6114**	5. Is affectionate to me	5374	0.82	0.95
**pf6117**	8. Confides closely in me	5376	0.62	0.95
**pf6119**	10. Understands my problems and worries	5373	0.67	0.95
**pf6122**	13. Is physically gentle and considerate	5367	0.75	0.95
**pf6123**	14. Makes me feel needed	5374	0.83	0.95
**pf6125**	16. Is very loving to me	5370	0.84	0.94
**pf6127**	18. Is fun to be with	5377	0.76	0.95
**pf6130**	21. Show his/her appreciation of me	5358	0.79	0.95
**pf6132**	23. Is gentle and kind to me	5378	0.85	0.94
**pf6133**	24. Speaks to me in a warm and friendly voice	5377	0.78	0.95
**Control subscale**				
**pf6111**	2. Wants me to take his/her side in an argument	5331	0.34	0.88
**pf6112**	3. Wants to know exactly what I'm doing and where I am	5372	0.41	0.88
**pf6115**	6. Is clearly hurt if I don't accept his/her views	5369	0.46	0.88
**pf6116**	7. Tends to try and change me	5369	0.69	0.86
**pf6118**	9. Tends to criticize me over small issues	5366	0.63	0.87
**pf6120**	11 Tends to order me about	5371	0.70	0.86
**pf6121**	12. Insists I do exactly as I'm told	5374	0.67	0.87
**pf6124**	15. Wants me to change in small ways	5372	0.51	0.88
**pf6126**	17. Seeks to dominate me	5374	0.71	0.86
**pf6128**	19. Wants to change me in big ways	5373	0.63	0.87
**pf6129**	20 Tends to control everything I do	5372	0.62	0.87
**pf6131**	22. Is critical of me in private	5323	0.56	0.87
**Total Cronbach’s alpha for Care and Control subscales were 0.95 and 0.88, respectively.**				

IBM: Intimate Bond Measure; ALSPAC: Avon Longitudinal Study of Parents and Children *It is measured only for those who lived with their partners when the age of index children was 2 years 9 months (pf questionnaire). **†**The number of non missing values of the item.

In the Control subscale there were three items that had relatively low correlations with the total subscale score: questions 2 (‘my partner wants me to take his/her side in an argument’), 3 (my partner wants to know exactly what I'm doing and where I am’), and 6 (‘my partner is clearly hurt if I don't accept his/her views’). As these three items have much weaker associations with the ‘control’ dimension, we therefore computed the Control subscale only for participants who completed all subscale items for the purpose of this Data Note. We recognise that this may introduce bias and other approaches such as multiple imputation should be considered. Results below are based on participants with data on at least six items for the Care subscale and all of the items for the Control subscale.

### Internal consistency

Table 3 & Table 4 show the internal consistency for mothers’ and partners’ Care and Control subscales at baseline. The Cronbach’s alpha was 0.95 for the Care and 0.88 for the Control subscales in both mothers and their partners. We repeated analyses and at all five timepoints and the same items, No. 8 in the Care (My partner confides closely in me) and No. 2 in the Control subscales (my partner wants me to take his/her side in an argument), had the lowest correlation with their respective subscales in both mothers and their partners (Supplemental Tables 3 & 4).

### Distributions of the Care and Control subscales

**Figure 1.**
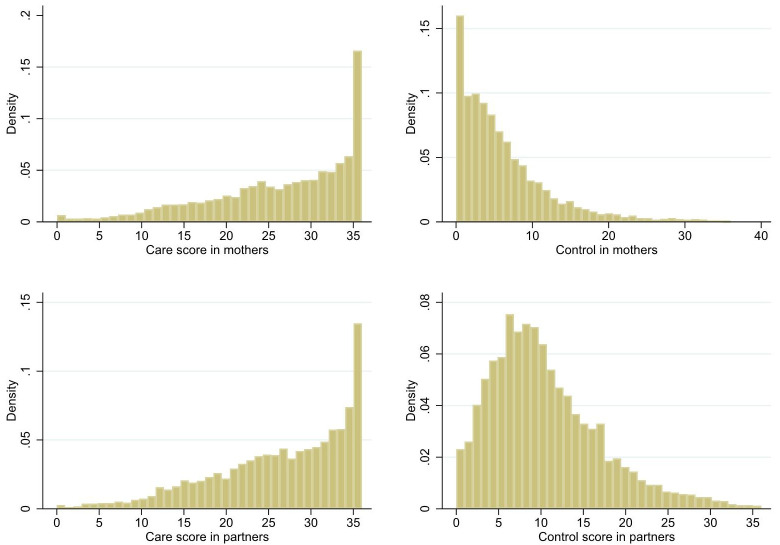
Distribution of IBM subscales in ALSPAC G0 parents at timepoint 1 (age of index children: 2 years 9 months). Top row: mothers (n = 8675); Bottom row: partners (n = 5499).

Figure 1 shows the distributions of the two subscales in both mothers and their partners at baseline, at mean ages 32.3±4.6 and 34.3±5.6, respectively. Care scores were skewed to the left at all time points while the Control subscale distributions were skewed to the right with a tendency toward a more normal distribution in men but not in women.

Basic descriptive data on the two subscales over the five timepoints for mothers and partners are shown in Table 5. Despite our differing approaches to calculating the subscales, this does not result in a large loss of data for the Control subscale. At timepoint 3 (9 years 2 months post-partum) we did not compute Control scores for 5.5% of mothers who had Care scores. This was the timepoint with the largest proportion of participants for whom we did not calculate a Control subscale score due to missing data on items with a low correlation with the total subscale score.

**Table 5.** Descriptive statistics of the Intimate Bond Measure (IBM) subscales in G0 mothers and partners in ALSPAC.

G0 mothers					
Time points (age of index children)	Mean Age ± SD	Care score		Control score	
		Sample size	Median (1 ^st^-3 ^rd^ quartile)	Sample size	Median (1 ^st^-3 ^rd^ quartile)
**2 years 9 months**	32.27±4.59	8568	28 (21-33)	8213	5 (2-9)
**6 years 1 month**	35.25±4.49	7431	28 (20-33)	7134	4 (2-8)
**9 years 2 months**	38.47±4.49	6818	28 (20-34)	6442	4 (2-8)
**12 years 1 month**	41.49±4.44	5870	28 (20-34)	5635	4 (2-8)
**18 years 5 months**	48.62±4.40	3311	30.6 (23-35)	3196	4 (2-7)
G0 partners					
Timepoints (age of index children)	Mean Age ± SD	Care score		Control score	
		Sample size	Median (1 ^st^-3 ^rd^ quartile)	Sample size	Median (1 ^st^-3 ^rd^ quartile)
**2 years 9 months**	34.29±5.63	5380	28 (21-34)	5207	9 (6-14)
**6 years 1 month**	37.84±5.56	3948	29 (21-34)	3834	9 (5-13)
**9 years 2 months**	41.32±5.47	3432	29 (21-34)	3335	8 (5-13)
**12 years 1 month**	44.18±5.52	3094	29 (22-34)	2991	8 (5-13)
**20 years 5 months**	53.30±5.36	2068	31 (24-35)	1987	8 (5-12)

SD: Standard deviations; The Care and Control subscales range between 0–36. The number of mothers who had the Care and Control scores at all time points were 2583 and 2210, respectively. The number of partners who had the Care and Control scores at all time points were 1009 and 888, respectively.

There was a moderate negative correlation between the Care and Control subscales at different timepoints (-0.37≤r≤-0.46) suggesting that high 'Care' is associated with less 'Control’ (Supplemental Table 5). These estimates are comparable to the results of a previous study ( Wilhelm & Parker, 1988). Distributions of the Care and Control scores in both mothers and their partners showed that men are more prone to report higher levels of Control by their partner than women.

Table 6 shows the number of couples with IBM data available for both partners at each time point based on our approach for calculation of subscale scores. This varied from 5,139 to 1,379 couples. The correlations are moderately high and consistent for the Care subscale (around 0.55) though dropping off for the 5 ^th^ time point (0.40) which was different for the men and women. The correlations for the Control subscale were far weaker (around 0.20) and again dropped off for the 5 ^th^ time point (0.11). 

**Table 6.** Number of couples with IBM data for both partners and the correlation of IBM subscales between couples at each timepoint in ALSPAC *.

Time points Subscales	2 years 9 months	6 years 1 month	9 years 2 months	12 years 1 month	18–20 years
**Care, Correlation (n)**	0.55 (5139)	0.56 (4182)	0.57 (3294)	0.58 (2913)	0.40 (1480)
**Control, Correlation (n)**	0.21 (4813)	0.21(3924)	0.19 (3049)	0.21 (2716)	0.11 (1379)

*It is measured only for those who lived with their partners.

### Associations between the IBM Care and Control subscales and socio-demographic characteristics

For both mothers and partners mean Care scores increased and Control scores decreased over time ( Table 5). We observed different patterns when we stratified the subscale scores by age group, educational level, occupation and parity ( Table 7 & Table 8 for mothers and partners respectively). Older mothers reported lower Care scores but this age-related pattern was not seen for partners. Higher educational level was associated with higher Care scores for both mothers and partners at baseline but over time these differences attenuated and were consistent with chance. Occupational social class showed even weaker gradients and a dose response effect (p-value for trend =0.01was observed for baseline only in mothers). Similar effects were seen with parity so that lower parity was associated with better Care scores at baseline for both mothers and partners but this attenuated over time.

**Table 7.** Distribution of the Care subscale score in different subgroups of mothers and partners in ALSPAC.

	Mothers					Partners				
Time points (postpartum)	2 years 9 months	6 years 1 month	9 years 2 months	12 years 1 month	18–20 years	2 years 9 months	6 years 1 month	9 years 2 months	12 years 1 month	18–20 years
**Age group † (years)**										
<25	26.10±8.39	26.41±8.70	26.48±8.81	26.95±8.57	28.52±8.05	26.63±8.15	26.86±8.02	27.10±8.47	27.29±8.31	29.61±6.27
25–30	26.24±8.30	26.14±8.67	26.06±8.76	26.07±8.92	27.98±8.23	26.70±8.05	26.84±8.15	26.95±8.36	27.20±8.13	28.29±8.26
30–35	26.15±8.46	25.67±8.93	25.50±9.01	25.36±9.23	27.20±8.45	26.54±8.10	26.82±8.24	26.42±8.46	26.73±8.36	28.69±7.74
35–40	25.32±8.97	24.59±9.33	25.14±9.16	24.32±9.18	25.80±9.09	26.77±8.11	26.63±8.62	26.57±8.65	26.69±8.53	28.95±7.21
>40	24.05±9.16	22.74±9.42	23.15±10.02	21.93±10.27	22.27±9.42	27.01±8.59	27.47±8.50	26.85±9.00	27.24±8.13	28.44±7.50
Test for trend (p-value)	0.21	<0.001	<0.001	<0.001	<0.001	0.41	0.36	0.54	0.45	0.55
**Education**										
CSE	26.01±8.78	26.20±9.17	26.38±9.31	26.18±9.53	27.32±8.65	26.14±8.80	27.02±8.38	27.59±8.48	26.69±9.00	28.39±8.73
Vocational	25.44±8.67	25.95±8.97	26.20±9.11	25.80±9.34	27.35±8.74	26.17±8.35	27.15±8.05	25.84±9.12	27.40±8.11	28.91±7.26
O level	25.81±8.49	25.57±8.98	25.73±8.94	25.74±9.14	27.87±8.43	25.89±8.36	26.38±8.46	26.38±8.50	27.01±8.42	28.68±7.71
A level	26.21±8.27	25.83±8.67	25.80±8.77	25.92±8.64	27.51±8.39	26.84±8.01	26.57±8.46	26.49±8.74	27.20±8.16	28.61±7.76
Degree	27.10±7.74	26.46±8.31	25.95±8.51	26.04±8.72	27.61±8.09	27.35±7.52	27.11±7.96	26.85±8.01	26.61±8.19	28.35±7.64
Test for trend (p-value)	0.006	0.49	0.007	0.11	0.49	0.003	0.64	0.09	0.15	0.13
**Occupational social class**										
I & II (Professional, Managerial & technical)	26.46±8.27	26.23±8.53	25.78±8.76	25.71±8.92	27.67±8.39	27.09±7.71	27.13±7.90	26.99±8.13	27.03±8.11	28.99±7.39
IIINM (Skilled non-manual)	25.42±8.47	24.88±9.09	25.35±8.98	25.49±8.79	27.78±8.42	26.32±8.01	25.93±8.61	25.72±9.02	26.63±8.52	28.18±8.20
IIIM Manual or IV partly skilled or V unskilled	25.92±8.61	26.39±8.62	25.91±9.04	26.63±8.57	27.02±8.54	26.71±8.21	26.81±8.17	26.53±8.67	27.14±8.26	28.13±8.15
Test for trend (p-value)	0.01	0.38	0.88	0.23	0.58	0.29	0.27	0.31	0.62	0.10
**Parity**										
0	26.60±8.16	26.10±8.64	26.09±8.76	26.27±8.70	27.62±8.43	27.10±7.77	27.05±8.11	27.02±8.33	27.1±8.13	28.63±7.77
1	25.93±8.36	26.10±8.77	25.89±8.87	25.95±8.99	27.94±8.14	26.29±8.33	26.77±8.33	26.60±8.49	27.12±8.34	28.60±7.60
2+	25.52±8.92	25.37±9.25	25.78±8.99	24.91±9.57	27.15±8.60	25.96±8.86	26.49±8.62	26.32±8.84	26.23±8.77	28.06±8.28
test for trend	<0.001	0.18	0.41	0.002	0.35	0.003	0.29	0.13	0.28	0.36

† Age group at delivery of the index children for mothers (variable: mz028b) and age in pregnancy for partners (pa910).

**Table 8.** Distribution of the Control subscale score in different subgroups of mothers and partners in ALSPAC.

	Mothers					Partners				
Time points (postpartum)	2 years 9 months	6 years 1 month	9 years 2 months	12 years 1 month	18–20 years	2 years 9 months	6 years 1 month	9 years 2 months	12 years 1 month	18–20 years
**Age group † (years)**										
<25	7.07±6.13	6.31±5.94	5.67±5.75	5.28±5.45	4.84±5.19	10.95±6.95	9.94±6.7	9.40±6.58	9.42±6.56	9.28±6.33
25–30	6.17±5.71	5.84±5.63	5.67±5.77	5.55±5.87	5.17±5.23	10.27±6.64	9.44±6.39	9.11±6.46	8.96±6.51	8.94±6.33
30–35	6.35±5.81	6.12±5.88	5.95±5.91	5.97±5.96	5.50±5.63	10.40±6.78	9.81±6.35	9.51±6.57	9.79±6.38	9.10±6.05
35–40	6.99±6.33	6.62±6.55	6.45±6.46	6.28±6.41	6.12±6.13	10.13±6.70	10.01±6.91	9.99±7.30	10.05±7.29	10.01±6.50
>40	9.26±8.31	9.32±8.35	9.68±7.70	9.78±9.01	8.62±8.28	10.16±7.29	9.77±6.49	9.75±7.18	9.89±7.13	10.13±6.75
Test for trend	0.15	0.56	0.001	<0.001	<0.001	0.06	0.36	0.20	0.03	0.03
**Education**										
CSE	6.53±6.14	5.75±6.06	5.81±6.34	5.48±5.93	5.21±6.21	9.74±7.15	8.87±7.00	8.20±6.81	8.87±7.06	8.75±6.58
Vocational	6.62±6.23	5.87±5.72	5.23±5.73	4.881±5.54	4.97±5.95	9.77±6.94	8.96±6.41	8.56±6.67	7.76±6.80	7.97±6.21
O level	6.48±5.88	6.09±5.87	5.74±5.86	5.71±6.10	5.11±5.47	10.48±6.90	9.75±6.41	9.25±6.57	8.92±6.34	8.80±6.20
A level	6.38±5.72	6.28±5.79	5.99±5.89	5.72±5.64	5.32±5.15	10.12±6.57	9.35±6.38	9.33±6.65	9.15±6.29	8.93±6.18
Degree	6.32±5.58	6.17±5.67	5.96±5.44	5.97±5.82	5.75±5.37	11.10±6.48	10.92±6.51	10.56±6.51	10.82±6.81	9.96±6.36
Test for trend (p-value)	0.88	<0.001	<0.001	<0.001	<0.001	<0.001	<0.001	<0.001	<0.001	<0.001
**Occupational social class**										
I & II (Professional, Managerial & technical)	6.44±5.99	6.21±5.93	5.92±6.00	5.76±5.82	5.28±5.38	10.54±6.39	10.36±6.52	9.84±6.56	9.85±6.45	9.20±6.24
IIINM (Skilled non-manual)	6.31±5.63	5.97±5.83	5.56±5.74	5.45±5.71	4.99±5.35	10.50±6.77	9.54±6.12	9.05±6.17	9.15±6.06	8.81±5.95
IIIM Manual or IV partly skilled or V unskilled	7.04±6.48	6.1±5.91	5.90±5.97	5.57±5.56	5.32±5.36	9.70±6.84	8.92±6.60	8.68±6.54	8.49±6.60	8.41±5.91
Test for trend (p-value)	0.13	0.35	0.66	0.34	0.49	<0.001	<0.001	<0.001	<0.001	0.06
**Parity**										
0	6.39±5.72	5.89±5.63	5.59±5.60	5.41±5.51	5.11±5.25	10.48±6.79	9.98±6.70	9.44±6.56	9.47±6.50	9.16±6.37
1	6.29±5.75	6.12±5.82	5.89±5.84	5.68±5.95	5.36±5.36	10.21±6.65	9.71±6.42	9.33±6.45	9.38±6.73	9.16±6.22
2+	7.07±6.53	6.57±6.38	6.12±6.37	6.32±6.58	5.67±5.99	10.45±6.96	9.21±6.27	9.55±7.36	9.71±7.15	9.56±6.58
Test for trend	0.055	0.01	0.10	0.004	0.07	0.62	0.03	0.50	0.82	0.36

† Age group at delivery of the index children for mothers (variable: mz028b) and age in pregnancy for partners (pa910).

For the Control subscale, we found more complex patterns. Age group initially showed a J-shape for mothers (worse (i.e. higher) Control score for younger and older mothers) but over time became worse for older mothers though that increase appeared more exponential. Mean Control scores in partners showed a much more modest increase with age. Higher educational level was associated with worse (i.e. higher) scores for both mothers and partners. Social class showed no obvious patterns for the mothers but for partners higher occupational social class was associated with worse (i.e. higher) scores. Higher parity was associated with worse Control scores in mothers but not in partners.

## Considerations when using the QoR data

Missing data is important to consider when using the IBM in ALSPAC. It could lead to selection bias if the loss to follow-up is related to both the exposure and the outcome depending on how the scores are being used ( Hernán *et al.*, 2004). Of note, we found no evidence of differences between participants who did and did not complete all IBM related items amongst those who returned the questionnaires. However, researchers should consider any direct or indirect association between loss to follow-up and the exposures and outcomes of interest in their analysis plan as well as an appropriate approach to address missing data such as multiple imputation ( Spratt *et al.*, 2010) or inverse probability-of-censoring weights ( Cain & Cole, 2009; Robins & Finkelstein, 2000). Some participants did not complete the IBM items because they did not have a partner at some timepoints for various reasons e.g. partner death or a relationship break-up. This is informative when studying QoR and will likely need to be accounted for in studies using these data.

We used two different approaches to generate the Care and Control subscale scores based on the correlation structures of their items. However, other researchers using these data may wish to handle the data items and derive scores using a different approach. At this stage we have not compared different methods for score derivation but any statistical analysis plan should pre-specify what will be done as the primary analysis and any sensitivity analyses using different methods.

The IBM is not the only measure that has been used in ALSPAC to measure QoR. There are a range of additional measures that could be used ( http://www.bristol.ac.uk/alspac/researchers/our-data/), for example: affection score (d369), aggression score (d375), emotional support (g217), satisfaction with partner (h572), rows with partner score (h583), activities together score (h590), communication between mother and partner score (h601) in mothers, and affection score (pa369), aggression score (pa372), sexual attitude (pc323), and sexual relationship (fa7100-7108), etc. in partners.

## Strengths and limitations of the data

One of the strengths of this data is the repeated measures of IBM on five occasions which can enable researchers to assess trajectories of QoR over time and consider the entire trajectory as an exposure or outcome. IBM is a validated scale with both Care and Control components, which allows a more detailed examination of QoR in a large sample size. Another advantage of this data is that it has been measured in both partners which made it possible for users to assess the correlation and/or discordance between mothers and their partners’ reports over time as well as predictors/outcomes of that discordance. The longitudinal nature of the data has made it a reliable source to assess causal relationship between QoR and various exposures, mediators, or outcomes avoiding many biases and reducing the risk of reverse causality.

One of the most important limitations for using IBM data in ALSPAC is that unlike mothers who are fixed over time, partners could change during follow-up. Therefore, when assessing the trajectory of IBM in mothers and/or partners, researchers may wish to consider additional information on partner changes which has recently been added to the ALSPAC data ( Northstone *et al.*, 2023). In addition, all included couples were eligible for inclusion due to the ALSPAC index pregnancy. This is important as couples who have never had a pregnancy are excluded, hence there are issues of generalizability as well as potential for selection bias.

## Ethical approval and consent

Ethical approval for the study was obtained from the ALSPAC Law and Ethics Committee (ALEC; IRB00003312) and the local research ethics committees. Informed consent for the use of data collected via questionnaires and clinics was obtained from participants following the recommendations of the ALSPAC Ethics and Law Committee at the time. Questionnaires were completed in the participants’ own home and return of the questionnaires was taken as continued consent for their data to be included in the study ( Birmingham & Golding, 2018). Full details of the approvals are available from the study website. Study members have the right to withdraw their consent for elements of the study or from the study entirely at any time.

## Data Availability

ALSPAC data access is through a system of managed open access. The steps below highlight how to apply for access to the data included in this data note and all other ALSPAC data. The datasets presented in this article are linked to ALSPAC project number B3929, please quote this project number during your application. The ALSPAC variable codes highlighted in the dataset descriptions can be used to specify required variables. 1. Please read the ALSPAC access policy which describes the process of accessing the data and samples in detail, and outlines the costs associated with doing so. 2. You may also find it useful to browse our fully searchable research proposals database, which lists all research projects that have been approved since April 2011. 3. Please submit your research proposal for consideration by the ALSPAC Executive Committee. You will receive a response within 10 working days to advise you whether your proposal has been approved. If you have any questions about accessing data, please email alspac-data@bristol.ac.uk. The study website also contains details of all the data that is available through a fully searchable data dictionary. OSF: Quality of relationships in mothers and their partners in the Avon Longitudinal Study of Parents and Children. https://doi.org/10.17605/OSF.IO/XQV7C ( Tohidinik *et al., 2023*). This project contains the following extended data: Create IBM scores.do (Stata code to create summary scores for IBM subscales, Stata file). Supplemental Tables for QoR.pdf SAGER_checklist QoR.pdf (the completed SAGER checklist). Data are available under the terms of the Creative Commons Attribution 4.0 International license (CC-BY 4.0).

## References

[ref-1] Bennett-Britton I Teyhan A Macleod J : Changes in marital quality over 6 years and its association with cardiovascular disease risk factors in men: findings from the ALSPAC prospective cohort study. *J Epidemiol Community Health.* 2017;71(11):1094–1100. 10.1136/jech-2017-209178 28993473 PMC5847094

[ref-2] Berkman LF Syme SL : Social networks, host resistance, and mortality: a nine-year follow-up study of Alameda County residents. *Am J Epidemiol.* 1979;109(2):186–204. 10.1093/oxfordjournals.aje.a112674 425958

[ref-3] Birmingham K Golding J : Pioneering ethics in a longitudinal study: The early development of the ALSPAC Ethics and Law Committee.Bristol: Public Policy Press,2018. Reference Source

[ref-4] Boyd A Golding J Macleod J : Cohort Profile: the 'children of the 90s'--the index offspring of the Avon Longitudinal Study of Parents and Children. *Int J Epidemiol.* 2013;42(1):111–127. 10.1093/ije/dys064 22507743 PMC3600618

[ref-5] Cain LE Cole SR : Inverse probability‐of‐censoring weights for the correction of time‐varying noncompliance in the effect of randomized highly active antiretroviral therapy on incident AIDS or death. *Stat Med.* 2009;28(12):1725–1738. 10.1002/sim.3585 19347843

[ref-6] Eder SJ Nicholson AA Stefanczyk MM : Securing your relationship: Quality of intimate relationships during the COVID-19 pandemic can be predicted by attachment style. *Front Psychol.* 2021;12:647956. 10.3389/fpsyg.2021.647956 34366966 PMC8334360

[ref-7] Fraser A Macdonald-Wallis C Tilling K : Cohort Profile: the Avon Longitudinal Study of Parents and Children: ALSPAC mothers cohort. *Int J Epidemiol.* 2013;42(1):97–110. 10.1093/ije/dys066 22507742 PMC3600619

[ref-8] Frech A Williams K : Depression and the psychological benefits of entering marriage. *J Health Soc Behav.* 2007;48(2):149–163. 10.1177/002214650704800204 17583271

[ref-9] Golding J Pembrey M Jones R : ALSPAC--the Avon Longitudinal Study of Parents and Children. I. Study methodology. *Paediatr Perinat Epidemiol.* 2001;15(1):74–87. 10.1046/j.1365-3016.2001.00325.x 11237119

[ref-10] Hernán MA Hernández-Díaz S Robins JM : A structural approach to selection bias. *Epidemiology.* 2004;15(5):615–625. 10.1097/01.ede.0000135174.63482.43 15308962

[ref-11] Hernán MR Robins JM : Causal Inference: What If.Boca Raton: Chapman & Hall/CRC Press,2020. Reference Source

[ref-12] Holt-Lunstad J Birmingham W Jones BQ : Is there something unique about marriage? The relative impact of marital status, relationship quality, and network social support on ambulatory blood pressure and mental health. *Ann Behav Med.* 2008;35(2):239–244. 10.1007/s12160-008-9018-y 18347896

[ref-13] Howe LD Tilling K Galobardes B : Loss to follow-up in cohort studies: bias in estimates of socioeconomic inequalities. *Epidemiology.* 2013;24(1):1–9. 10.1097/EDE.0b013e31827623b1 23211345 PMC5102324

[ref-23] Major-Smith D Heron J Fraser A : The Avon Longitudinal Study of Parents and Children (ALSPAC): a 2022 update on the enrolled sample of mothers and the associated baseline data [version 1; peer review: awaiting peer review]. *Wellcome Open Res.* 2022;7:283. 10.12688/wellcomeopenres.18564.1 37664415 PMC10472060

[ref-24] Northstone K Ben Shlomo Y Teyhan A : The Avon Longitudinal Study of Parents and children ALSPAC G0 Partners: A cohort profile [version 1; peer review: awaiting peer review]. *Wellcome Open Res.* 2023;8:37.

[ref-14] Northstone K Lewcock M Groom A : The Avon Longitudinal Study of Parents and Children (ALSPAC): an update on the enrolled sample of index children in 2019 [version 1; peer review: 2 approved]. *Wellcome Open Res.* 2019;4:51. 10.12688/wellcomeopenres.15132.1 31020050 PMC6464058

[ref-15] Pieh C O' Rourke T Budimir S : Relationship quality and mental health during COVID-19 lockdown. *PLoS One.* 2020;15(9):e0238906. 10.1371/journal.pone.0238906 32915878 PMC7485771

[ref-16] Robins JM Finkelstein DM : Correcting for noncompliance and dependent censoring in an AIDS clinical trial with inverse probability of censoring weighted (IPCW) log‐rank tests. *Biometrics.* 2000;56(3):779–788. 10.1111/j.0006-341x.2000.00779.x 10985216

[ref-17] Rothman KJ : Epidemiology. An introduction.New York: Oxford University Press,2002.

[ref-18] Spratt M Carpenter J Sterne JA : Strategies for multiple imputation in longitudinal studies. *Am J Epidemiol.* 2010;172(4):478–487. 10.1093/aje/kwq137 20616200

[ref-19] Taylor SE : Social support: A review.In: Friedman HS (ed.), *The Oxford handbook of health psychology*. Oxford University Press,2011;189–214. Reference Source

[ref-20] Tohidinik HR Ben-Shlomo Y Smith D : Quality of relationships in mothers and their partners in the Avon Longitudinal Study of Parents and Children.[Dataset].2023. 10.17605/OSF.IO/XQV7C PMC10594051 37881255

[ref-21] Uchino BN : Social support and health: a review of physiological processes potentially underlying links to disease outcomes. *J Behav Med.* 2006;29(4):377–387. 10.1007/s10865-006-9056-5 16758315

[ref-22] Wilhelm K Parker G : The development of a measure of intimate bonds. *Psychol Med.* 1988;18(1):225–234. 10.1017/s0033291700002051 3363041

