# Supplementary material for: Quality of relationships in mothers and their partners in the Avon Longitudinal Study of Parents and Children
Source: Wellcome Open Res. 2023 Oct 11;8:62. Originally published 2023 Feb 6. [Version 2] doi: 10.12688/wellcomeopenres.18835.2 (PMC10594051; doi:10.12688/wellcomeopenres.18835.2)
Supplement: Peer review history

# Reviewer report: Reviewer response for version 2

**DOI:** 10.21956/wellcomeopenres.22365.r68436

**Published:** 2023-10-23

**Authors:** Todd M Jensen

**Affiliations:** 1 The University of North Carolina at Chapel Hill, Chapel Hill, North Carolina, USA

I believe the authors have been responsive to the comments offered during the first round of reviews. I am satisfied with their revisions, and I have no further comments at this point.

Are sufficient details of methods and materials provided to allow replication by others?

Partly

Is the rationale for creating the dataset(s) clearly described?

Yes

Are the datasets clearly presented in a useable and accessible format?

Yes

Are the protocols appropriate and is the work technically sound?

Partly

Reviewer Expertise:

Family relationship dynamics and their associations with individual well-being over time; psychometrics; advanced quantitative methodology

I confirm that I have read this submission and believe that I have an appropriate level of expertise to confirm that it is of an acceptable scientific standard.

# Reviewer report: Reviewer response for version 2

**DOI:** 10.21956/wellcomeopenres.22365.r68435

**Published:** 2023-10-19

**Authors:** Alexandre A Lussier

**Affiliations:** 1 Harvard Medical School, Harvard University, Boston, Massachusetts, USA

I am satisfied with the efforts of the authors to revise this manuscript. I have no further comments.

Are sufficient details of methods and materials provided to allow replication by others?

Yes

Is the rationale for creating the dataset(s) clearly described?

Yes

Are the datasets clearly presented in a useable and accessible format?

Yes

Are the protocols appropriate and is the work technically sound?

Yes

Reviewer Expertise:

Early-life environments; mental health; genetics; epigenetics; longitudinal studies

I confirm that I have read this submission and believe that I have an appropriate level of expertise to confirm that it is of an acceptable scientific standard.

# Reviewer report: Reviewer response for version 1

**DOI:** 10.21956/wellcomeopenres.20882.r63987

**Published:** 2023-09-07

**Authors:** Alexandre A Lussier

**Affiliations:** 1 Harvard Medical School, Harvard University, Boston, Massachusetts, USA

This data note provides an overview of the Intimate Bond Measure (IBM) used to assess the quality of mother-partner relationships in the ALSPAC cohort. The IBM is reported five times between the time the child was age 2.75 to 18 from both the mother and her partner, and consists of two primary scales measuring "Care" and "Control". 

 The authors found that both scales had high internal reliability, though the Control scale showed some questions with lower reliability and correlations. These findings suggest that missing values in the two scales should be interpreted and accounted for with caution. The authors also provide descriptive statistics between the Care and Control scales and key demographic variables in the cohort, including maternal age, education, occupational social class, and parity.

 Overall, this is a well-written and informative data note that provides good insight into the strengths and limitations of the IBM. I have a few suggestions and minor comments to help readers better assess and contextualize this measure within the ALSPAC dataset. 

 Introduction

 1. It is unclear whether any prior studies using ALSPAC data have analyzed the IBM itself. It would be helpful to reference these (if any), or to state directly that no prior studies have used it. 

 Results

 2. In the first paragraph of "Distributions of the Care and Control subscales", please state to which age these results refer. It would also be helpful to include the distributions of the other ages in the supplemental materials as a reference. 

 3. Please consider including graphs to show the results from the demographic analyses in the supplemental materials, as these would make the data structures being described easier to understand and visualize. 

 4. Similarly, it is possible that some of the patterns identified are due to changes in partners across time, something to which the authors allude in the discussion. It would be helpful to provide accompanying statistics on how the partners of mother change over time, as these are likely a major driver of differences for the IBM measures at different timepoints. 

 Minor points

 5. Table 1 has a cell formatting error in Has a partner, 6 years 1 months, partners - the cells for male and female partner have been merged. 

 6. In Figure 1, it is strange that x-axis of the top-right panel goes to 40, when the rest end at 35 (and the scale is on a 0-36 range). 

 7. Please include the timepoint in the titles of Tables 3 and 4.

Are sufficient details of methods and materials provided to allow replication by others?

Yes

Is the rationale for creating the dataset(s) clearly described?

Yes

Are the datasets clearly presented in a useable and accessible format?

Yes

Are the protocols appropriate and is the work technically sound?

Yes

Reviewer Expertise:

Early-life environments; mental health; genetics; epigenetics; longitudinal studies

I confirm that I have read this submission and believe that I have an appropriate level of expertise to confirm that it is of an acceptable scientific standard, however I have significant reservations, as outlined above.

# Reviewer report: Reviewer response for version 1

**DOI:** 10.21956/wellcomeopenres.20882.r62026

**Published:** 2023-08-11

**Authors:** Todd M Jensen

**Affiliations:** 1 The University of North Carolina at Chapel Hill, Chapel Hill, North Carolina, USA

I reviewed the Data Note entitled “Quality of relationships in mothers and their partners in the Avon Longitudinal Study of Parents and Children.” Overall, the manuscript was well written and the rationale for the general focus on measures of couple relationship quality over time was well reasoned. Below I have provided some comments that are intended to inform efforts to bolster the data note.

 1) When assessing internal consistency reliability, consider use of McDonald’s Omega as a supplement to Cronbach’s alpha. Alpha imposes relatively stringent assumptions on the data. See the following reference:

 McNeish, D. (2018). Thanks coefficient alpha, we’ll take it from here. *Psychological methods*, *23*(3), 412-433.

 2) Did the authors consider use of factor analytic models and full information maximum likelihood to handle missing data? Factor analysis would enable robust assessment of item weights and other psychometric features that could be useful. In the event missing data meet the assumption of Missing at Random (see work of Craig Enders and others), FIML might enable the use of significantly more cases. I do want to recognize that the authors have noted that this data note features one of many potential approaches to examining the measures of interest; however, I do think factor analysis could be a strong approach to consider.

 3) It would be helpful to provide details about the specific type of correlation statistics estimated. Given that the substantive items were technically ordinal, polychoric correlations are likely optimal.

 4) I’m not sure “external validity” is the appropriate term when discussing the rationale for assessing correlations between IBM subscale scores and various sociodemographic and socioeconomic variables.

 5) The authors note that Table 3 and Table 4 feature reliability information for key measures at baseline. Consider adding information to the table titles or notes that highlight the focus on the baseline period of data collection. Also, consider providing reliability results from the other time-points.

 6) Change “this data” to “these data” (p. 12 in “strengths and limitations of the data” subsection).

Are sufficient details of methods and materials provided to allow replication by others?

Partly

Is the rationale for creating the dataset(s) clearly described?

Yes

Are the datasets clearly presented in a useable and accessible format?

Yes

Are the protocols appropriate and is the work technically sound?

Partly

Reviewer Expertise:

Family relationship dynamics and their associations with individual well-being over time; psychometrics; advanced quantitative methodology

I confirm that I have read this submission and believe that I have an appropriate level of expertise to confirm that it is of an acceptable scientific standard, however I have significant reservations, as outlined above.

## References

[1] : Thanks coefficient alpha, we’ll take it from here. *Psychological Methods* .2018;23(3) : 10.1037/met0000144 412-433 10.1037/met0000144 28557467
